# Comparative Efficacy and Safety of Robot-Assisted vs. Freehand Screw Placement in Femoral Neck Fractures: An Updated Systematic Review and Meta-Analysis

**DOI:** 10.3390/jcm13175072

**Published:** 2024-08-27

**Authors:** Ümit Mert, Mohamad Agha Mahmoud, Maher Ghandour, Ahmad Al Zuabi, Marco Speicher, Philipp Kobbe, Klemens Horst, Frank Hildebrand, Koroush Kabir

**Affiliations:** 1 Department of Orthopedics and Trauma Surgery, Helios University Hospital, University Witten/Herdecke, 42283 Wuppertal, Germany; mghandourmd@gmail.com (M.G.); marco.speicher@helios-gesundheit.de (M.S.); koroush.kabir@helios-gesundheit.de (K.K.); 2 Department of Orthopedics, Trauma and Reconstructive Surgery, RWTH Aachen University, 52062 Aachen, Germany; m.agha.mahmoud@gmail.com (M.A.M.); ahmad.alzuabi@hotmail.com (A.A.Z.); khorst@ukaachen.de (K.H.); fhildebrand@ukaachen.de (F.H.); 3 Department of Trauma and Reconstructive Surgery, BG Bergmannstrost, 06120 Halle (Saale), Germany; philipp.kobbe@bergmannstrost.de; 4 Department of Trauma and Reconstructive Surgery, University Hospital Halle, 06120 Halle (Saale), Germany

**Keywords:** femoral neck fracture, robot-assisted screw placement, freehand technique, orthopedic surgery, systematic review, meta-analysis

## Abstract

**Background:** Femoral neck fractures pose significant surgical challenges with high morbidity and mortality. Traditional freehand screw placement often yields variable outcomes. Recent robotic advancements offer a promising alternative with enhanced precision. **Methods:** This systematic review compares the efficacy and safety of robot-assisted versus freehand techniques. A comprehensive literature search across multiple databases up to July 2024 included studies comparing both techniques. Primary outcomes were the union rate and time, functional outcomes, operative time, intraoperative parameters, and complication rates. Meta-regression analyses identified treatment response determinants. **Results:** Twenty-four studies (1437 patients) were included. Robot-assisted screw placement significantly improved the union rate, reduced the union time, and showed superior functional outcomes. Additionally, it resulted in shorter operative times, less intraoperative blood loss, and fewer instances of fluoroscopy and guide pin insertion. The risk of femoral neck necrosis was notably lower with robotic assistance. Meta-regression highlighted the robot type, patient age, and sample size as significant factors. **Conclusions:** Despite the promise of robot-assisted screw placement, limitations exist. The evidence being mainly from China raises concerns about generalizability. The lack of long-term follow-up data hinders assessment of technique durability. Unreported surgeon expertise levels and learning curves affect result validity. High initial costs and steep learning curves of robotic systems also present barriers to widespread adoption.

## 1. Introduction

Femoral neck fractures are among the most common and severe injuries, particularly in the elderly population, often leading to significant morbidity and mortality [1]. These injuries usually result from low-energy trauma, particularly in osteoporotic patients [2]. These fractures pose a substantial challenge to orthopedic surgeons due to the complex anatomical and biomechanical characteristics of the femoral neck, as well as the high risk of complications such as avascular necrosis (disruption of the vascular supply, thus compromising the healing process) and nonunion [3]. Effective and timely surgical intervention (proper screw placement) is crucial for improving patient outcomes, restoring function, and reducing the length of the hospital stay [4]. The inaccurate positioning of screws compromises the mechanical stability, leading to implant failure and, subsequently, reoperation [5]. This can also impact postoperative rehabilitation, prolonging recovery time and increasing healthcare costs [6].

Recently, robotic-assisted systems have become popular due to their enhanced precision, achieved through computer-aided positioning and real-time imaging. Traditional freehand screw placement has been the standard approach for the surgical management of femoral neck fractures [7]. While this technique is well established and widely used, it is associated with several limitations, including variability in screw placement accuracy, long operative times, increased intraoperative blood loss, and higher radiation exposure due to repeated fluoroscopy [8]. These factors can contribute to suboptimal outcomes and increased risk of complications.

In recent years, advancements in robotic technology have introduced robot-assisted screw placement as a promising alternative to the conventional freehand technique [9]. Robotic systems are supposed to enhance precision, stability, and control during surgery, potentially overcoming the limitations associated with manual screw placement. By providing real-time imaging and navigation, robotic assistance might have the potential to improve the accuracy of screw placement, reduce operative time, minimize blood loss, and lower radiation exposure [10]. These advantages suggest that robot-assisted screw placement could lead to better clinical outcomes and higher patient satisfaction. In addition to traditional methods, robot-assisted techniques should be compared to other minimally invasive approaches. Techniques such as arthroscopic surgery and percutaneous pinning, while less technologically advanced, offer reduced recovery times and minimal soft tissue disruption [11]. However, robotic systems provide an additional level of precision and control that may enhance outcomes even further.

Despite the theoretical benefits of robotic systems, the clinical evidence supporting their use in femoral neck fracture management remains varied and, in some cases, inconclusive. Previous studies have reported mixed results, and comprehensive evaluations comparing the efficacy and safety of robot-assisted versus freehand screw placement are limited [9,12,13,14,15,16]. However, conflicting results in the literature about the efficacy of robotic-assisted techniques stem from several key factors. Variability in surgeon experience, differences in patient populations, and the heterogeneity of robotic systems used in different studies contribute to inconsistent findings. Moreover, the limited availability of long-term data has made it difficult to draw definitive conclusions about the superiority of robotic assistance. A previous systematic review was conducted showing the superiority of the robot-assisted approach [10]. However, since then, numerous studies have been conducted, showing conflicting conclusions [17,18,19,20,21,22,23]. To address this gap in the literature, our updated systematic review and meta-analysis aims to critically assess and synthesize the available evidence on the comparative outcomes of these two surgical techniques.

This study will consider various clinical outcomes, including union rate, union time, functional scores, operative time, intraoperative parameters, and complication rates. Additionally, we will explore potential determinants of treatment response based on meta-regression analyses to provide deeper insights into the factors influencing the effectiveness of robot-assisted screw placement. Through this rigorous evaluation, we aim to provide robust evidence to guide clinical decision-making and optimize the management of femoral neck fractures.

## 2. Materials and Methods

### 2.1. Study Design

This systematic review and meta-analysis was conducted following the Preferred Reporting Items for Systematic Reviews and Meta-Analyses (PRISMA) guidelines, which were updated in 2020 [24]. This review protocol was not registered on PROSPERO prior to the commencement of this work given the quick turnaround time of this work.

### 2.2. Literature Search

To identify eligible studies, PubMed, Scopus, Web of Science, Cochrane Central Register of Controlled Trials (CENTRAL), clinicaltrials.gov, and Google Scholar (only the first 200 citations were selected) [25], were searched from inception to 9 July 2024 using a combination of keywords and medical subject heading (MeSH) terms, including “robot”, “femoral neck”, and “fracture”. The search strategy, outlined in Table S1, was adjusted for each database accordingly. Citations were filtered based on their titles and abstracts. No restrictions were applied regarding the original language of publication. To ensure the accuracy of the performed search and screening, we searched for relevant studies manually by reading the reference list of finally selected papers by checking the list of “similar articles” to selected ones on PubMed and by manually searching Google software using the same keywords included in the literature search [26].

### 2.3. Selection Strategy

The eligibility criteria were based on the refined PICOS (Population, Intervention, Comparison, Outcomes, and Study Design) framework [27]. Selected studies followed the following criteria:

1. Experimental (randomized or non-randomized trial) and observation studies (study design).
2. Studies including patients with femoral neck fracture (population).
3. Patients receiving robot-assisted screw placement (intervention).
4. Studies comparing a robotic approach to a freehand approach (comparison/control group).
5. Studies reporting one of our outcomes of interest (listed in the next sub-section).

On the other hand, studies meeting the following criteria were excluded:

1. Non-original research (i.e., reviews, editorials, perspectives, commentaries, etc.).
2. Published protocols without results.
3. Studies without a comparison group.
4. Validation studies reporting the feasibility of implementing robotics in orthopedic surgery.
5. Irrelevant populations (patients without femoral neck fracture or fracture at other sites).
6. No clear description of the intervention or comparison groups.
7. Duplicates studies.
8. Studies with overlapping patients’ data.

### 2.4. Data Collection and Outcome Measures

The senior author designed the data-collection sheet using Microsoft Excel. The sheet was divided into three sections. The first one contained data pertaining to included studies (authors’ names, country, year of publication, study design, and follow-up), examined patients (sample size, age, gender), and allocated groups (type of robotic method used). The second part contained data related to our outcomes of interest. These included union time and rate, intraoperative fluoroscopy time and frequency, Harris score (high values indicate greater improvement), operative time, intraoperative blood loss, frequency of guide pin insertion, postoperative hospitalization time, frequency of intraoperative drilling, frequency of needle position adjustment, and postoperative complications. The third section covered the risk of bias assessment of the included studies.

Investigators were blinded to studies’ country and year of publication, as well as authors’ names to minimize the risk of judgment bias. Each study was given an ID in this regard, which was cross-validated with the original study title by the corresponding author. Other descriptive data of the included studies were removed (i.e., authors’ names, country/year of investigation, study design). Two authors were blinded to other investigators’ work, and their role was to ensure the accuracy of the extracted data. In instances where inaccurate data or inconsistent reporting was found, a meeting with the corresponding author was carried out to correct these mistakes.

### 2.5. Risk of Bias Assessment

The risk of bias (RoB) of the included randomized controlled trials was examined using the 2019 revised Cochrane RoB-2 tool. Each RCTs was assessed in terms of several aspects: randomization, deviations from intended interventions, missing outcome data, and the selection of reported results. Each domain is given a rating of either “low risk”, “high risk”, or “some concerns” [28]. Overall, if a study had high risk in one domain, it was designated as having an overall high risk of bias. If a study had a low risk in all domains, it was designated as having an overall low risk of bias. Otherwise, the study was designated with an overall rating of some concerns. Meanwhile, for cohort studies, the ROBINS-I tool for non-randomized studies of intervention was used. In this tool, 7 domains are assessed, including confounding, selection, classification of interventions, deviations from intended interventions, missing outcome data, outcome measurement, and selective reporting. An overall grade of low, moderate, or high risk was given.

### 2.6. Statistical Analysis

All statistical analyses were carried out using STATA Software (Version 18, Stata Corp, College Station, TX, USA). For continuous outcomes (i.e., union time, fluoroscopy time, etc.), the mean difference (MD) and its corresponding 95% confidence interval (CI) was calculated. Meanwhile, for binary outcomes (i.e., union rate), the odds ratio (OR) and its 95% CI were calculated. The statistical model was selected based on observed heterogeneity, measured by tau-square and I-square measures, with values > 40% and an associated *p* value < 0.05 defining significant heterogeneity [29]. If significant heterogeneity was observed, the random-effect model was selected; otherwise, the fixed-effect model was prioritized. The restricted maximum likelihood method (REML) method was implemented if heterogeneity was noted.

Leave-one-out sensitivity analyses were performed if significant heterogeneity was encountered to determine the change in the reported effect estimate following the exclusion of each of analyzed studies individually. Galbraith plots were inspected for outliers (none were identified). The potential for publication bias was assessed using funnel plots and formal tests for funnel plot asymmetry (Egger’s test and Begg’s rank correlation test) [30]. This was only feasible if the measured outcome was reported by at least 10 studies. We found no risk of publication bias.

Outcomes reported by 5 or more studies [31] were included in the meta-regression. Meta-regression was performed to investigate the impact of study-level covariates on the variation in measured outcomes. The covariates included both continuous (e.g., mean age, follow-up, and sample size) and categorical (e.g., robot type) variables, with dummy variables created for the latter [TiRobot was selected as the reference group]. A reference group for the creation of dummy variables was chosen based on the largest sample size category to provide a stable comparison group. Multicollinearity between covariates was assessed using variance inflation factors (VIFs), with a threshold VIF > 5 indicating problematic multicollinearity [32]. Covariates with high multicollinearity were excluded from the regression model (none were found). The adjusted R-squared will be used to assess the proportion of variance in the dependent variable that is predictable from the independent variables, adjusting for the number of predictors included in the model. A higher adjusted R-squared value, while adding covariates, indicates a better-fitting model with higher performance.

## 3. Results

### 3.1. Literature Search Results

**Figure 1:**
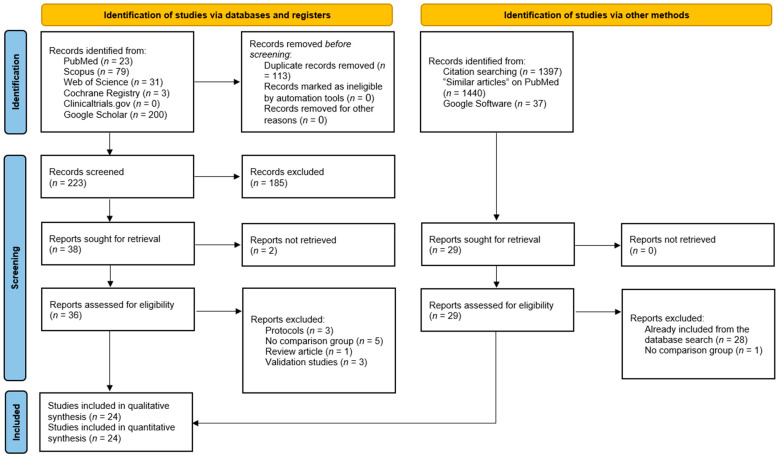
PRISMA diagram showing the results of the literature search and screening process.

We retrieved 336 hits from the database search, of which 113 duplicates were ruled out by EndNote Software (Figure 1). The initial screening of 223 records yielded 38 articles eligible for full-text screening. Two studies were unretrievable, and from the remaining 36 studies, 12 were excluded for the following reasons: protocols (*n* = 3), lack of a comparison group (*n* = 5), review article (*n* = 1), and validation studies (*n* = 3). The manual search did not add any additional studies, resulting in 24 studies eligible for data synthesis and analysis [12,13,14,16,17,18,19,20,21,22,23,33,34,35,36,37,38,39,40,41,42,43].

### 3.2. Baseline Characteristics of Included Studies

A summary of the characteristics of include studies is provided in Table 1. All of the included studies were conducted in China, most of which were retrospective cohort studies (22 studies), with only 2 randomized controlled trials. A total of 1437 patients with femoral neck fractures were examined, of whom 676 were in the robot-assisted group, and the remaining 761 patients were assigned to the freehand screw placement group. The follow-up period ranged from 6 months to as high as 38.8 months. Patients’ age ranged from 41.95 years to 71.3 years.

### 3.3. Risk of Bias of Included Studies

**Figure 2:**
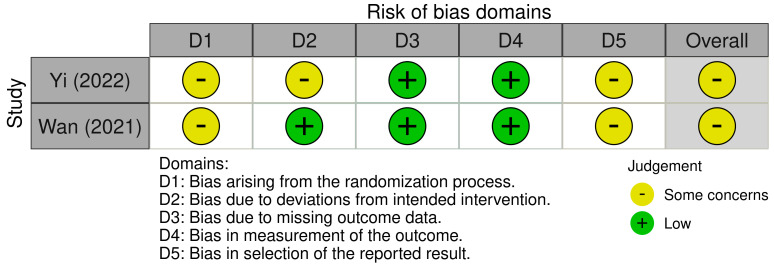
A summary of the risk of bias of randomized controlled trials using the revised version of the Cochrane tool (2019) [40,41].

The risk of bias of the two included randomized trials was graded as “some concern” (Figure 2). This was mainly attributed to the lack of a prior protocol and lack of information regarding the randomization process.

**Figure 3:**
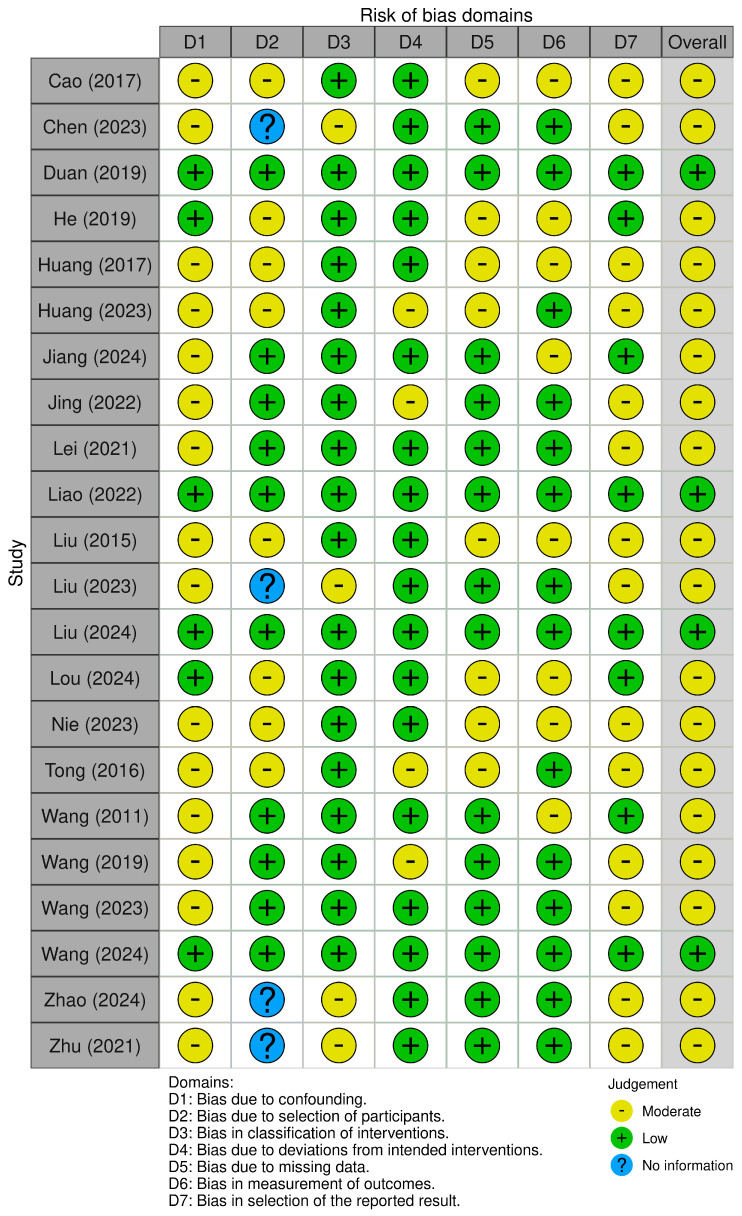
A summary of the risk of bias of nonrandomized studies of intervention using the ROBINS-I risk-of-bias tool [12,13,14,15,16,17,18,19,20,21,22,23,33,34,35,36,37,38,39,41,42,43].

As for the 22 cohort studies, only 4 studies had low risk of bias, while the remaining 18 studies had moderate risk of bias (Figure 3). This was mainly due to the lack of confounding control in their analyses and the uncertainty of their reporting secondary to the lack of a prior registered protocol.

### 3.4. Union Rate

**Figure 4:**
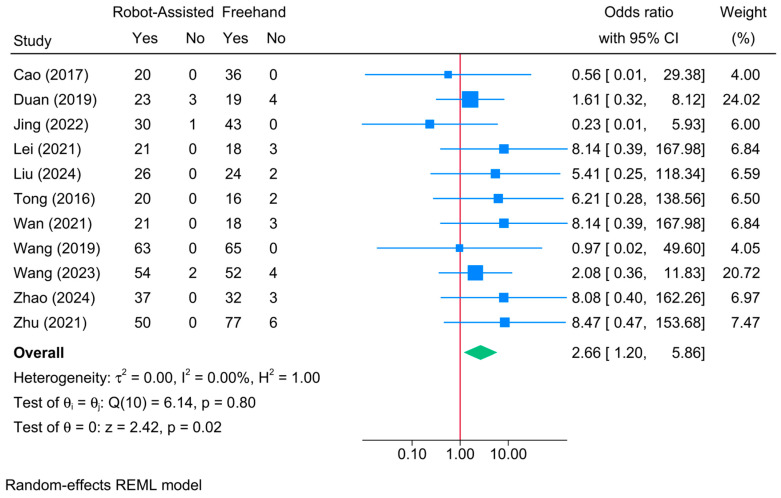
Forest plot showing the difference in union rate between robot-assisted and conventional freehand screw placement in femoral neck fractures [12,14,16,19,22,33,35,36,39,41,42].

Robot-assisted screw placement resulted in significantly higher odds of union compared to the conventional freehand technique [11 studies, OR = 2.66; 95% CI: 1.20–5.86] (Figure 4). No heterogeneity was observed (τ^2^ = 0; I^2^ = 0%, *p* = 0.80).

The meta-regression showed that patients’ age, sample size, follow-up period, and robot type were not determinants of union rate (Table 2).

### 3.5. Union Time (Days)

**Figure 5:**
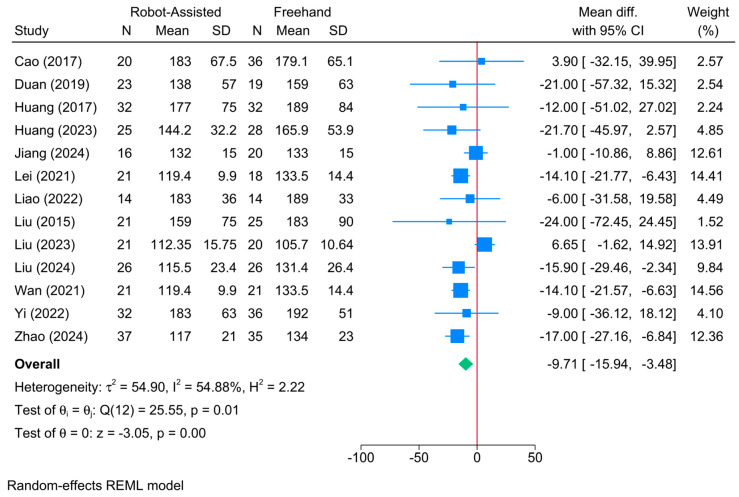
Forest plot showing the difference in union time between robot-assisted and conventional freehand screw placement in femoral neck fractures [12,18,19,33,34,36,37,38,39,40,42,43].

A significantly shorter union time was observed in the robot-assisted screw placement group compared to the conventional freehand technique [13 studies, MD = −9.71; 95% CI: −15.94: −3.48] (Figure 5). A moderate level of heterogeneity was observed (τ^2^ = 54.90; I^2^ = 54.88%, *p* = 0.01). However, the sensitivity analysis revealed no change in the reported estimate (Figure S1).

The meta-regression showed that sample size was the only determinant of treatment effect (coefficient = −0.351; *p* = 0.043) (Table 2). The model fit was perfect with no residual heterogeneity (R^2^ = 100%; residual I^2^ = 0%)

### 3.6. Harris Score

**Figure 6:**
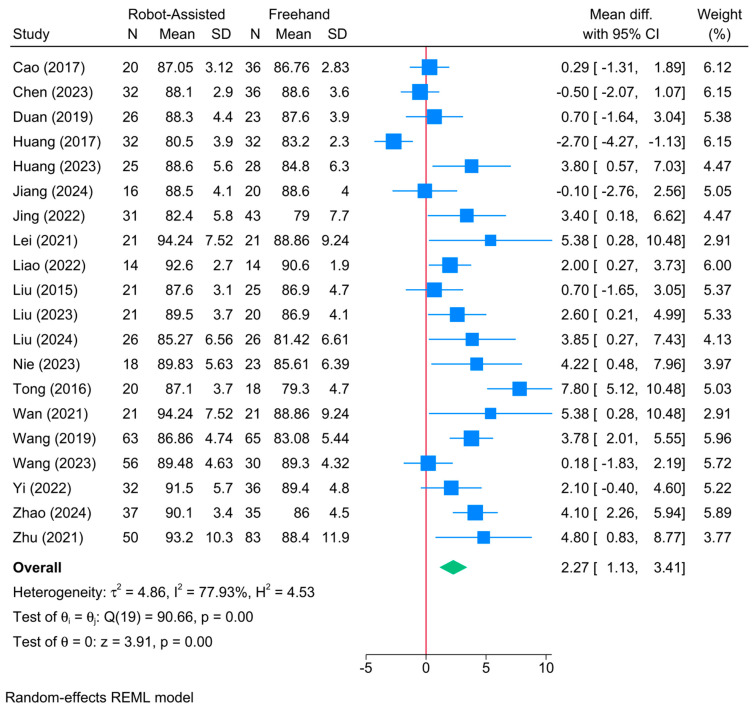
Forest plot showing the difference in Harris score between robot-assisted and conventional freehand screw placement in femoral neck fractures [12,14,16,17,18,19,21,22,33,34,35,36,37,38,39,40,41,42,43].

A significant improvement in the Harris score was observed in the robot-assisted screw placement group compared to the conventional freehand technique [20 studies, MD = 2.27; 95% CI: 1.13–3.41] (Figure 6). A high level of heterogeneity was observed (τ^2^ = 4.86; I^2^ = 77.93%, *p* = 0.001). However, the sensitivity analysis revealed no change in the reported estimate (Figure S2).

The meta-regression showed that none of analyzed factors were significant determinants of Harris score (*p* > 0.05) (Table 2). Model fit was poor with a high degree of residual heterogeneity (R^2^ = 0%; residual I^2^ = 78.86%)

### 3.7. Operative Time (Mins)

**Figure 7:**
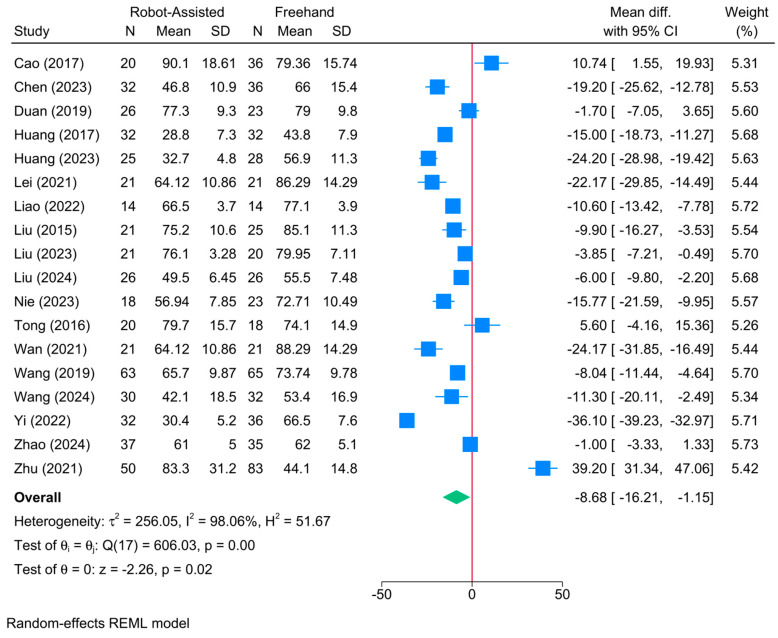
Forest plot showing the difference in operative time between robot-assisted and conventional freehand screw placement in femoral neck fractures [12,14,16,17,19,21,23,33,34,36,37,38,39,40,41,42,43].

The robot-assisted screw placement group resulted in a significantly shorter operative period compared to the conventional freehand technique [18 studies, MD = −8.68; 95% CI: −16.21: −1.15] (Figure 7). A high level of heterogeneity was observed (τ^2^ = 256.05; I^2^ = 98.06%, *p* = 0.001). The sensitivity analysis revealed no difference between both groups following the exclusion of the study of Yi et al. [41] (Figure S3).

The meta-regression showed that robot type was the only determinant of treatment effect (Table 2). Compared to TiRobot, bi-planar robots (coefficient = −20.47; *p* = 0.0001) were associated with a greater reduction in operative time. However, the model fit was poor with a high degree of residual heterogeneity (R^2^ = 33.43%; residual I^2^ = 97.13%).

### 3.8. Frequency of Fluoroscopy

**Figure 8:**
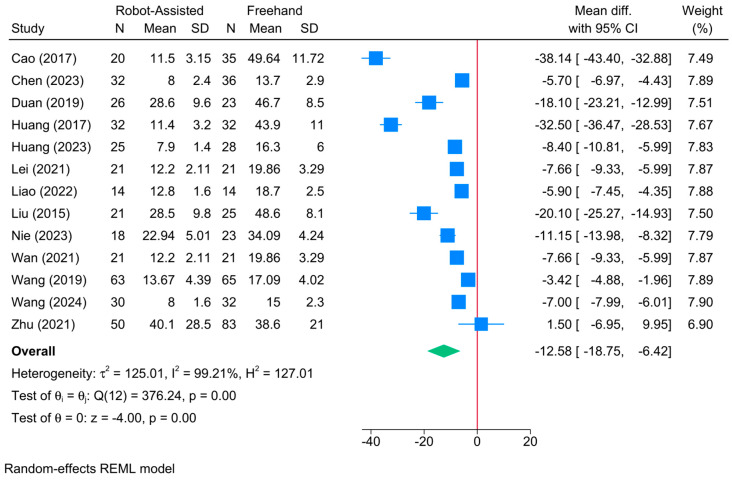
Forest plot showing the difference in the frequency of fluoroscopy time between robot-assisted and conventional freehand screw placement in femoral neck fractures [12,16,17,21,23,33,34,36,37,38,39,41,43].

The robot-assisted screw placement group was associated with a lower frequency of fluoroscopy compared to the conventional freehand technique [13 studies, MD = −8.68; 95% CI: −16.21: −1.15] (Figure 8). A high level of heterogeneity was observed (τ^2^ = 125.01; I^2^ = 99.21%, *p* = 0.001). However, the sensitivity analysis revealed no change in the reported estimate (Figure S4).

The meta-regression showed that robot type and patients’ age significantly predicted treatment effect (Table 2). Compared to TiRobot, both bi-planar (coefficient = −20.47; *p* = 0.0001) and universal robots (coefficient = −32.17; *p* = 0.0001) were associated with a greater reduction in the frequency of fluoroscopy. A unit increase in patients’ age reduced the frequency of fluoroscopy by 0.45 (*p* = 0.01). Model fit was perfect but with a high degree of residual heterogeneity (R^2^ = 94.67%; residual I^2^ = 85.30%).

### 3.9. Intraoperative Fluoroscopy Time (s)

**Figure 9:**
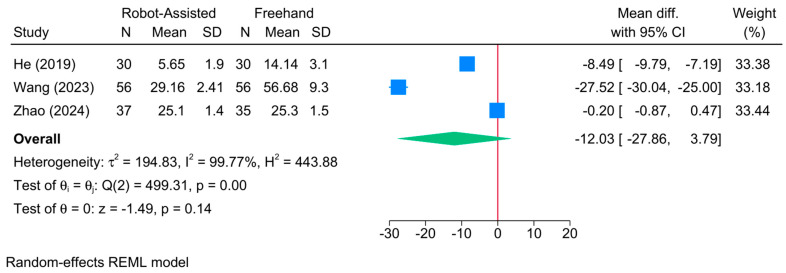
Forest plot showing the difference in intraoperative fluoroscopy time between robot-assisted and conventional freehand screw placement in femoral neck fractures [13,22,42].

No significant difference was found between the robot-assisted screw placement and the conventional freehand groups in terms of intraoperative fluoroscopy time [3 studies, MD = −12.03; 95% CI: −27.86: 3.79] (Figure 9). A high level of heterogeneity was observed (τ^2^ = 194.83; I^2^ = 99.77%, *p* = 0.001). However, the sensitivity analysis revealed no change in the reported estimate (Figure S5). Due to the small sample, meta-regression analysis was not feasible.

### 3.10. Frequency of Guide Pin Insertion

**Figure 10:**
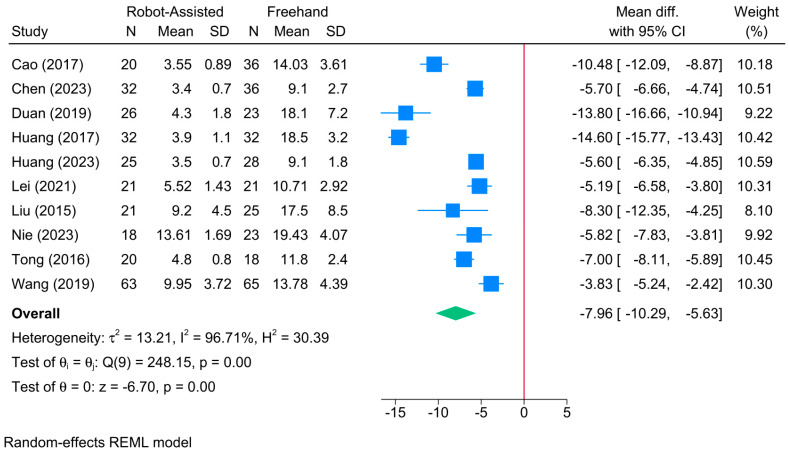
Forest plot showing the difference in frequency of guide pin insertion between robot-assisted and conventional freehand screw placement in femoral neck fractures [12,14,16,17,21,33,34,36,38,43].

The robot-assisted screw placement group was associated with a lower frequency of guide pin insertion compared to the conventional freehand technique [10 studies, MD = −7.96; 95% CI: −10.29: −5.63] (Figure 10). A high level of heterogeneity was observed (τ^2^ = 13.21; I^2^ = 96.71%, *p* = 0.001). However, the sensitivity analysis revealed no change in the reported estimate (Figure S6).

The meta-regression showed that only the robot type significantly predicted the treatment effect (Table 2). Compared to TiRobot, universal robots (coefficient = −7.28; *p* = 0.045) were associated with a greater reduction in the frequency of guide pin insertion. The model fit was fair with a high degree of residual heterogeneity (R^2^ = 44.45%; residual I^2^ = 92.81%).

### 3.11. Frequency of Intraoperative Drilling

**Figure 11:**
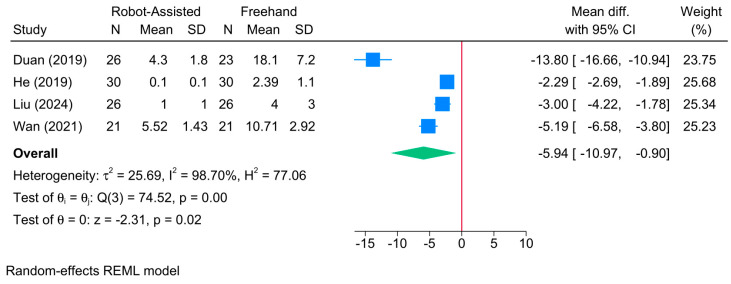
Forest plot showing the difference in frequency of intraoperative drilling between robot-assisted and conventional freehand screw placement in femoral neck fractures [12,13,19,39].

The robot-assisted screw placement group was associated with a lower frequency of intraoperative drilling compared to the conventional freehand technique [4 studies, MD = −5.94; 95% CI: −10.97: −0.90] (Figure 11). A high level of heterogeneity was observed (τ^2^ = 25.69; I^2^ = 98.70%, *p* = 0.001). However, the sensitivity analysis revealed no significant difference between both groups following the exclusion of the study of Wan et al. [40] (Figure S7). Due to the small sample, meta-regression analysis was not feasible.

### 3.12. Frequency of Guide Pin Adjustment

**Figure 12:**
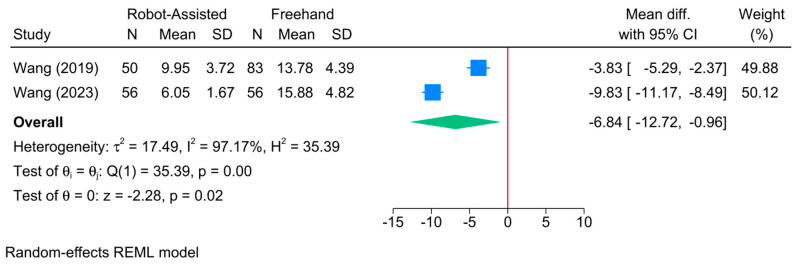
Forest plot showing the difference in frequency of the needle position adjustment between robot-assisted and conventional freehand screw placement in femoral neck fractures [16,22].

The robot-assisted screw placement group was associated with a lower frequency of guide pin adjustment compared to the conventional freehand technique [2 studies, MD = −6.84; 95% CI: −12.72: −0.96] (Figure 12). A high level of heterogeneity was observed (τ^2^ = 17.49; I^2^ = 97.17%, *p* = 0.001). Due to the small sample, conducting sensitivity and meta-regression analyses was not feasible.

### 3.13. Intraoperative Blood Loss (mL)

**Figure 13:**
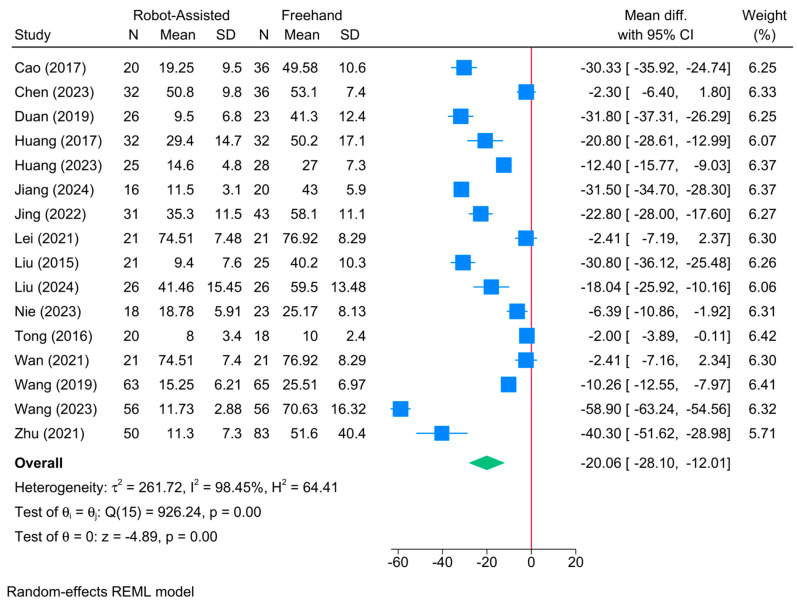
Forest plot showing the difference in intraoperative blood loss between robot-assisted and conventional freehand screw placement in femoral neck fractures [12,14,16,17,18,19,21,22,33,34,35,36,38,39,41,43].

The robot-assisted screw placement group was associated with a lower volume of intraoperative blood loss compared to the conventional freehand technique [16 studies, MD = −20.06; 95% CI: −28.10: −12.01] (Figure 13). A high level of heterogeneity was observed (τ^2^ = 261.72; I^2^ = 98.45%, *p* = 0.001). However, the sensitivity analysis revealed no change in the reported estimate (Figure S8).

The meta-regression showed that robot type, sample size, and patients’ age significantly modified treatment effect (Table 2). Compared to TiRobot, Universal robots (coefficient = −37.71; *p* = 0.007) were associated with a greater reduction in intraoperative blood loss. Also, sample size (coefficient = −0.225; *p* = 0.035) and mean age (coefficient = −2.98; *p* = 0.0001) were inversely associated with blood loss. Model fit was fair with a high degree of residual heterogeneity (R^2^ = 51.16%; residual I^2^ = 96.49%).

### 3.14. Postoperative Hospitalization Time (Day)

**Figure 14:**
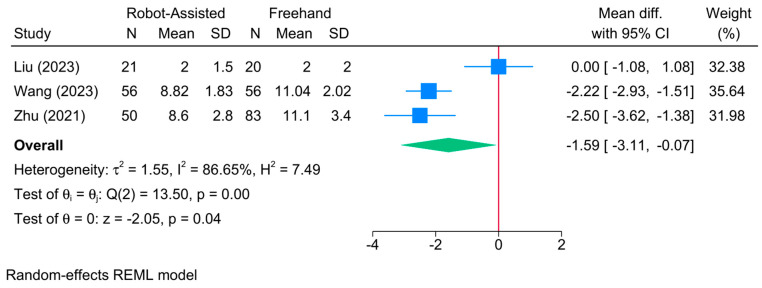
Forest plot showing the difference in postoperative hospitalization time between robot-assisted and conventional freehand screw placement in femoral neck fractures [22,39,41].

The robot-assisted screw placement group was associated with a lower postoperative hospitalization time compared to the conventional freehand technique [3 studies, MD = −1.59; 95% CI: −3.11: −0.07] (Figure 14). A high level of heterogeneity was observed (τ^2^ = 1.55; I^2^ = 86.65%, *p* = 0.001). However, the sensitivity analysis revealed a significant variability in the reported estimate (Figure S9). Due to the small sample, meta-regression analysis was not feasible.

### 3.15. Postoperative Complications

**Figure 15:**
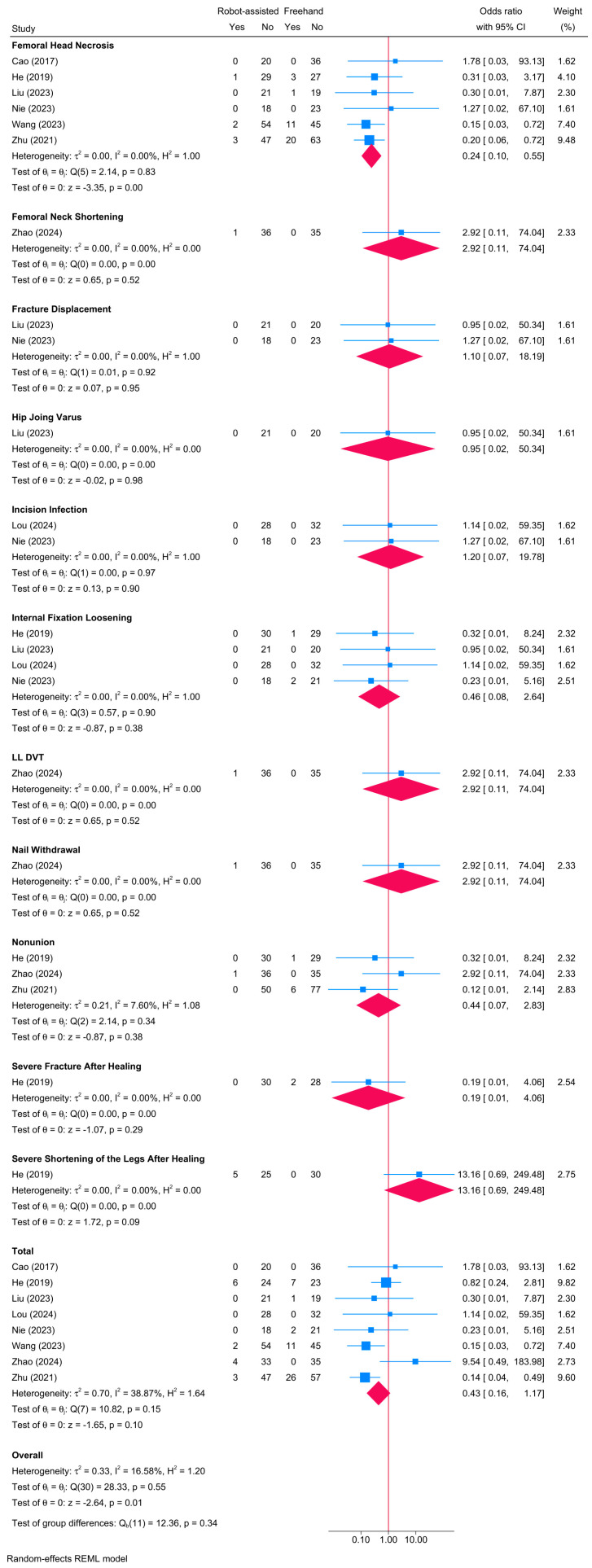
Forest plot showing the difference in postoperative complications between robot-assisted and conventional freehand screw placement in femoral neck fractures [13,20,21,22,33,39,41,42].

Regarding overall complications, no significant difference was observed between the robot-assisted screw placement and the conventional freehand groups [8 studies, OR = 0.43; 95% CI: 0.16–1.17] (Figure 15). Heterogeneity was insignificant (τ^2^ = 0.70; I^2^ = 38.87%, *p* = 0.15). Noteworthy, the risk of femoral neck necrosis was significantly lower in the robot-assisted group compared to the freehand screw placement group [OR = 0.24; 95% CI: 0.10–0.55]. However, no differences were observed between both groups regarding femoral neck displacement, hip joint varus, incision infection, internal fixation loosening, lower limb deep vein thrombosis, nail withdrawal, nonunion, severe fracture post-healing, and severe leg shortening post-healing. Due to the variability in reported complications, a meta-regression analysis was not deemed informative.

## 4. Discussion

This systematic review and meta-analysis reveal the superior efficacy of robot-assisted screw placement over the conventional freehand technique for femoral neck fractures. Robot-assisted procedures significantly improved several key clinical outcomes, including union rate, union time, Harris score, operative time, frequency of fluoroscopy, guide pin insertion, intraoperative drilling, needle position adjustment, intraoperative blood loss, and postoperative hospitalization time. Additionally, the risk of femoral neck necrosis was notably reduced in the robot-assisted group.

### 4.1. Accuracy of Screw Placement

Robot-assisted procedures have demonstrated significant enhancements in precision due to advanced imaging systems and real-time navigation tools. These technologies enable a higher degree of control over screw placement, resulting in reduced malposition rates and optimized biomechanical stability within the femoral neck [9]. In contrast, freehand techniques, while requiring less infrastructure and relying heavily on the surgeon’s experience, often yield variable results. Inaccuracies in screw placement via the freehand method can contribute to postoperative complications, such as hardware failure and nonunion [45]. Thus, the advantages of robotic assistance in ensuring precise screw placement may outweigh those of conventional approaches, enhancing overall patient outcomes.

In our study, the robot-assisted technique significantly increased the union rate and decreased the union time. These findings can be attributed to the enhanced precision of robotic systems, which ensure optimal screw placement, thereby promoting better fracture stabilization and faster recovery [46]. The lack of heterogeneity in union rate underscores the consistency of this benefit across studies. The robot-assisted group exhibited significantly improved Harris scores, reflecting better functional outcomes. The high level of heterogeneity in this outcome suggests variability in patient characteristics, surgical techniques, and postoperative care protocols. However, the consistent improvement across studies highlights the overall effectiveness of the robotic approach in enhancing patient mobility and reducing pain.

### 4.2. Surgical Time and Efficiency

Surgical efficiency is crucial for both patient outcomes and operating room workflow. Studies have shown that robotic systems can reduce intraoperative time due to their precision and enhanced visualization capabilities [12,17,20,33,34,38,39]. However, freehand techniques may allow for quicker adaptation to unforeseen surgical challenges, though this flexibility can also lead to increased variability in surgical time. Balancing the technological advantages of robotic systems with the adaptability of traditional methods is essential, particularly in high-stakes environments where both surgical time and patient safety are paramount. Comprehensive analysis of these factors contributes to best practices in femoral neck screw placement.

In our study, robot-assisted procedures significantly reduced operative time and intraoperative blood loss. The precision of robotic systems likely minimizes tissue damage and bleeding, contributing to these outcomes [47]. The reduction in operative time also lowers anesthesia duration, further decreasing perioperative risks [48]. The robot-assisted group showed a significant reduction in the frequency of fluoroscopy and guide pin insertion. These findings indicate a decrease in radiation exposure and surgical complexity, benefiting both patients and surgical staff.

### 4.3. Postoperative Complications and Recovery

The robotic-assisted approach has been associated with a reduction in common postoperative complications such as infections, screw misplacement, and postoperative pain, which can delay rehabilitation and extend hospital stays [49]. The lower infection rate in the robot-assisted group can be attributed to the shorter surgical time facilitated by robotic systems. Shorter operative times reduce the duration of tissue exposure and the risk of contamination, which are critical factors in minimizing postoperative infections. Freehand techniques, while traditionally effective, may have higher variability in outcomes due to human factors and anatomical complexities. Understanding the implications of these complications is crucial for optimizing recovery.

In our analysis, the reduced postoperative hospitalization time in the robot-assisted group suggests faster recovery and fewer postoperative complications. However, the certainty of this finding is limited and is secondary to the high heterogeneity, small sample size, and wide confidence interval (−3.11 to −0.07) close to the null value. Although the overall complication rates did not differ significantly between the groups, the reduced risk of femoral neck necrosis highlights a crucial advantage of robotic assistance in preserving bone vascularity and integrity. The lower risk of necrosis observed in the robot-assisted group is likely attributable to the enhanced precision of screw placement afforded by robotic systems. This precision minimizes the risk of secondary displacement, which can compromise vascular integrity, leading to necrosis. Moreover, the reduced drilling time associated with robotic assistance may further contribute to preserving bone vascularity, thereby reducing the risk of avascular necrosis. Although our findings align with those previously published systematic reviews by highlighting the superiority of the robotic system over conventional screw placement [8,10], our study adds to available evidence by including a larger sample size, analyzing more outcomes, and by identifying significant predictors of treatment response.

### 4.4. Patient Outcomes and Satisfaction

Patient outcomes and satisfaction are critical metrics in evaluating surgical techniques. Robot-assisted surgeries often result in fewer misplacements and shorter recovery times, leading to higher patient satisfaction levels. Moreover, patients who feel more informed and involved in their treatment typically report greater satisfaction. None of the included studies examined these patient-reported outcomes.

### 4.5. Long-Term Functional Results

Long-term functional results are vital for understanding the ongoing impacts of surgical techniques on patient quality of life. Patients undergoing robot-assisted procedures often experience fewer complications and enhanced recovery trajectories due to precise screw placement, which promotes better bone healing and reduces the need for subsequent surgical interventions. Traditional freehand methods, while effective, may show variability in outcomes due to reliance on the surgeon’s skill and experience. Comprehensive follow-up assessments reveal that robotic assistance favorably influences long-term functional results, suggesting a paradigm shift in orthopedic surgical practices.

### 4.6. Determinants of Response Based on Meta-Regression

Our meta-regression analyses identified several determinants of treatment response, providing insights into the factors influencing the efficacy of robot-assisted screw placement. Different robotic systems exhibited varying levels of effectiveness. For instance, bi-planar robots significantly reduced operative time and frequency of fluoroscopy compared to TiRobot. Universal robots further decreased fluoroscopy frequency and intraoperative blood loss. These findings highlight the importance of selecting the appropriate robotic system tailored to specific surgical requirements. That being said, more research is still needed in order to run a network analysis of direct and indirect comparisons between different robotic systems to determine which one offers the best improvement in clinical outcomes.

Patient age and sample size also emerged as significant determinants. Older age was associated with a reduction in fluoroscopy frequency and intraoperative blood loss. Larger sample sizes predicted shorter union times and reduced blood loss, suggesting that larger studies may better capture the benefits of robotic assistance. This is logical as the larger the sample size, the more credible (higher confidence) the results become.

### 4.7. Surgeon’s Expertise

The proficiency of a surgeon in performing femoral neck screw placements significantly correlates with their experience and training, directly impacting surgical outcomes [12]. Extensive training in both traditional techniques and advanced robotic systems equips surgeons with a nuanced understanding of anatomical complexities and the dexterity required for precision. Research indicates that experienced surgeons are more likely to anticipate and mitigate potential complications, enhancing the safety and efficacy of operations [50]. Conversely, less experienced surgeons may require additional time to adapt to the technological intricacies of robot-assisted methods, potentially leading to increased operative time and variable outcomes [51]. The successful application of freehand techniques requires rigorous training that encompasses both theoretical knowledge and practical skill acquisition, while incorporating robotic-assisted techniques necessitates a well-structured training program to ensure proficiency and safety. The learning curve associated with each method influences the efficiency and outcomes, with robotic systems initially presenting a steep learning curve but ultimately providing improved precision and reduced intraoperative time as familiarity increases [52]. Surgeon confidence and skill levels significantly influence surgical outcomes, particularly in complex procedures like femoral neck screw placement, where technological advancements can boost confidence by providing enhanced visualization and precision. The correlation between a surgeon’s experience and surgical outcomes is critical, as increased proficiency leads to decreased operative times, reduced complication rates, and higher overall patient satisfaction, underscoring the importance of experience in achieving optimal results with both traditional and robotic techniques [53]. Unfortunately, none of included studies reported the level nor the learning curve of surgeons who performed these surgeries.

### 4.8. Cost Evaluation

Evaluating the economic implications of robotic assistance versus freehand techniques in femoral neck screw placement requires a comprehensive cost analysis. Robot-assisted surgery involves higher initial investments due to the cost of specialized equipment and training for surgical teams. However, innovations such as augmented reality integration can lead to long-term cost efficiencies through reduced postoperative complications and shorter recovery times [12,19,39]. In contrast, traditional freehand procedures incur fewer upfront costs but may result in increased rates of revision surgeries due to inaccuracies during implant placement, potentially elevating overall healthcare expenses [16,33]. The financial analysis must account for direct costs as well as potential savings from improved surgical outcomes, suggesting that the financial benefits of robotic assistance could outweigh the initial expenditures when considering long-term patient care costs. Unfortunately, there is no comprehensive evaluation of the costs associated with robotic screw placement in femoral neck fracture. Moreover, while the immediate financial implications of robotic systems are significant, a comprehensive evaluation of long-term benefits is crucial to understanding their overall cost-effectiveness. This nuanced perspective should also consider the impact on hospital resources and staffing, with robotic systems potentially improving surgical efficiency and reducing the need for revision surgeries, despite requiring specially trained personnel.

### 4.9. Implications for Clinical Practice

The integration of robotic-assisted technology in femoral neck screw placement has significant implications for clinical practice, particularly in enhancing surgical accuracy and patient outcomes. Robotic systems show substantial advantages in precision and surgical efficiency, reducing malposition rates and potentially decreasing postoperative complications such as nonunion or avascular necrosis. These benefits translate into shorter hospital stays and faster recovery times, which are critical in a healthcare environment focused on efficiency and cost-effectiveness.

The adoption of robotic systems requires a paradigm shift in surgical training and skill development, necessitating that clinicians embrace new methodologies to achieve proficiency in these innovative techniques. Robotic assistance is particularly beneficial in complex cases, offering intricate maneuverability and real-time feedback, which enhance the surgeon’s capabilities. However, the high initial cost and steep learning curve of robotic systems can be challenging for some institutions. Therefore, implementing robotic technology must balance clinical benefits with institutional readiness and capacity for change.

The findings of this study underscore the clinical superiority of robot-assisted screw placement for femoral neck fractures. The enhanced precision and control provided by robotic systems lead to better clinical outcomes, reduced operative complexity, and faster patient recovery. Significant reductions in operative time, intraoperative blood loss, and radiation exposure highlight the safety advantages of robotic assistance. Moreover, identifying key determinants of response can guide clinical decision-making and the selection of appropriate robotic systems, prompting a reevaluation of training programs and resource allocation to optimize patient care and surgical outcomes.

In practical terms, the meta-regression findings emphasize the need for a tailored approach in the adoption of robotic systems for femoral neck fracture surgeries. Clinicians should carefully select the appropriate robotic system based on its demonstrated effectiveness, particularly in younger patients or those undergoing multiple procedures. Additionally, the importance of surgeon training cannot be overstated; hospitals should ensure that their surgical teams are well-versed in the specific robotic technologies that offer the best outcomes. Finally, healthcare institutions should consider the cost-effectiveness of these systems, particularly in high-volume settings, to ensure that resource allocation maximizes patient benefits.

### 4.10. Study Limitations

The analysis in this study faces several limitations that require careful attention. First, the focus on quantitative metrics like postoperative complication rates and alignment accuracy may neglect qualitative aspects such as surgical experience and patient satisfaction. Second, the sample size, although statistically significant, lacks demographic diversity, limiting the applicability of the findings to broader populations. Specifically, all of the available evidence originated from China, suggesting that the robotic approaches are not yet validated in the Western world. Third, the absence of long-term follow-up data also hampers the assessment of the durability and effectiveness of robot-assisted techniques compared to traditional methods.

Fourth, the existing literature on this topic is limited and often presents conflicting results, complicating comparative interpretations. Additionally, some clinically important outcomes could not be meta-analyzed because they were reported by only one study. These included postoperative pain [39], hollow screw replacement time [18], guide needle drilling time [15], incision length [20], optimal blade position [19], and total placement time of cannulated screws [13]. Importantly, the frequency of repositioning was not described in the available evidence, except for one study, which defined the number of drilling attempts as the number of times the needle was repositioned during the insertion process [13]. This outcome needs to be further investigated in future research with a proper and standardized definition criterion.

Finally, none of examined evidence reported the expertise level and learning curves of performing surgeons, which could influence outcomes. The role of surgeon quality, including the experience levels of consultants or residents, was also not considered. A cost–benefit analysis was lacking in all studies; therefore, it was not synthesized in this review. Given these limitations, the conclusions may not fully capture the complexities of surgical practice and decision-making in femoral neck screw placement. Therefore, further research that includes qualitative analyses and diverse patient populations is needed to enhance the robustness of future findings.

### 4.11. Recommendations for Future Research

Future research in orthopedic surgery, particularly concerning femoral neck screw placement, should prioritize comprehensive comparative studies that evaluate not only surgical outcomes but also cost-effectiveness and patient recovery metrics. High-quality, multicenter randomized controlled trials with diverse populations are essential to confirm the current findings and enhance their applicability. Longitudinal assessments investigating the long-term effects of robot-assisted versus freehand techniques on postoperative complications, functional recovery, and quality of life could yield valuable insights, as current research primarily focuses on immediate outcomes. Expanding the diversity of study populations to include a wider range of age groups and comorbidities will enhance the generalizability of the results, providing a more nuanced understanding of how various demographics respond to different surgical methodologies. Additionally, qualitative research exploring surgeons’ perspectives on usability and educational requirements for adopting robotic systems could inform training protocols and facilitate smoother transitions into this technological realm. A multifaceted approach to future research will fortify the evidence base, guiding clinical decision-making in femoral neck screw placement and informing healthcare policy and practice.

## 5. Conclusions

This systematic review and meta-analysis provide compelling evidence that robot-assisted screw placement significantly improves clinical outcomes in patients with femoral neck fractures compared to the conventional freehand technique. The enhanced precision, reduced operative time, lower intraoperative blood loss, and reduced radiation exposure underscore the potential of robotic assistance as a transformative tool in orthopedic surgery. The identification of key determinants of response further refines our understanding of the factors influencing the effectiveness of robotic systems. These findings support the continued integration and advancement of robotic technologies in clinical practice to optimize patient outcomes and healthcare efficiency.

## Figures and Tables

**Table 1:** Summary description of the characteristics of included studies comparing robot-assisted to freehand screw placement in femoral neck fractures.

Author (YOP)	Country	Robot	Design	FU (mo)	Age		Sample			Gender (M/F)	
					RA	FH	RA	FH	Total	RA	FH
Cao (2017) [33]	China	Universal Robots	RCS	14.7	44.7	47.9	20	36	56	10/10	19/17
Chen (2023) [17]	China	TiRobot	RCS	31.4	43.6 (13.7)	45.7 (12.7)	32	36	68	18/14	17/19
Duan (2019) [12]	China	TiRobot	RCS	13.6	61.7 (5.2)	62.1 (4.1)	26	23	49	11/15	9/14
He (2019) [13]	China	Bi-planar robot	RCS	24	56 (11.1)	56.2 (9.13)	30	30	60	11/19	12/18
Huang (2017) [34]	China	Bi-planar robot	RCS	19.6	59.4 (5.6)	59.1 (4.9)	32	32	64	10/22	12/20
Huang (2023) [44]	China	TiRobot Advance	RCS	22.2	48.2 (11.9)	48.5 (9.8)	25	28	53	11/14	12/16
Jiang (2024) [18]	China	TiRobot	RCS	15.6	53.61 (5.45)	55.23 (4.64)	16	20	36	7/9	8/12
Jing (2022) [35]	China	TiRobot	RCS	7	55.2	55	31	43	74	11/20	14/29
Lei (2021) [36]	China	TiRobot	RCS	6	51.86 (4.89)	51.33 (4.3)	21	21	42	12/9	14/7
Liao (2022) [37]	China	TiRobot	RCS	8	44.1 (8.7)	48.8 (8)	14	14	28	6/8	7/7
Liu (2015) [38]	China	GD-2000	RCS	12.5	65.2 (4.2)	60.5 (5.1)	21	25	46	8/13	11/14
Liu (2023) [39]	China	TiRobot	RCS	24	71.3	73.2	21	20	41	10/11	8/12
Liu (2024) [19]	China	TiRobot	RCS	12	51.92 (6.41)	50.08 (8.41)	26	26	52	17-Sep	11/15
Lou (2024) [20]	China	Tianji Robot	RCS	13	46.2 (9.3)	48.2 (7.8)	28	32	60	12/16	15/17
Nie (2023) [21]	China	TiRobot	RCS	14.9	56 (4.22)	54.87 (4.81)	18	23	41	8/10	10/13
Tong (2016) [14]	China	TiRobot	RCS	18	47.5	51.5	20	18	38	12/8	11/7
Wan (2021) [40]	China	Tianji Robot	RCT	6	51.86 (4.89)	51.33 (4.3)	21	21	42	12/9	14/7
Wang (2011) [15]	China	Bi-planar robot	RCS	-	-	-	6	6	12	-	-
Wang (2019) [16]	China	TiRobot	RCS	12	49.03 (8.23)	49.8 (7.68)	63	65	128	30/33	31/34
Wang (2023) [22]	China	TiRobot	RCS	12	59.5 (8.7)	60.1 (8.2)	56	56	112	32/34	30/36
Wang (2024) [23]	China	TiRobot	RCS	6	41.95 (5.39)	40.8 (5.08)	30	32	62	-	-
Yi (2022) [41]	China	TINAVI	RCT	18	58.5 (6.3)	57.5 (5.3)	32	36	68	19/13	16/20
Zhao (2024) [43]	China	TiRobot	RCS	12	53.87 (5.28)	52.36 (5.05)	37	35	72	16/21	13/22
Zhu (2021) [42]	China	TiRobot	RCS	38.8	47.9 (13.5)	47.7 (12.6)	50	83	133	26/24	47/36

RCS: retrospective cohort study; RCT: randomized controlled trial; YOP: year of publication; FU: follow-up; mo: month; RA: robot-assisted; FH: freehand; M/F: male/female. Age data are presented as mean (standard deviation).

**Table 2:** A summary of the determinants of reported clinical outcomes through meta-regression analyses.

	Coefficient	SE	Z	P	Low CI	High CI
**Union rate**						
Universal Robots	−2.54082	2.304537	−1.1	0.27	−7.05764	1.975986
Tianji Robot	0.93305	1.775284	0.53	0.599	−2.54644	4.412544
Follow-up (month)	0.043397	0.065153	0.67	0.505	−0.0843	0.171095
Sample size	−0.00764	0.014116	−0.54	0.588	−0.03531	0.020026
Mean age (year)	−0.07405	0.089556	−0.83	0.408	−0.24958	0.101473
	R^2^ = 0%; I^2^ = 0%					
**Union Time (days)**						
Bi-planar robot	−0.902	12.397	−0.070	0.942	−25.199	23.395
Universal Robots	13.861	20.143	0.690	0.491	−25.620	53.341
GD-2000 Robot	−15.649	25.399	−0.620	0.538	−65.430	34.132
Tianji Robot	0.446	5.416	0.080	0.934	−10.169	11.062
TiRobot Advance	−21.236	16.661	−1.270	0.202	−53.890	11.418
Follow-up (month)	1.113	0.709	1.570	0.116	−0.277	2.503
Sample size	−0.351	0.173	−2.020	**0.043**	−0.690	−0.011
Mean age (year)	0.027	0.577	0.050	0.963	−1.105	1.159
	R^2^ = 100%; I^2^ = 0%					
**Harris Score**						
Bi-planar robot	−2.884	2.129	−1.350	0.175	−7.056	1.288
Universal Robots	−3.031	2.850	−1.060	0.288	−8.617	2.556
GD-2000	−1.587	3.096	−0.510	0.608	−7.654	4.481
Tianji Robot	2.153	3.778	0.570	0.569	−5.252	9.558
TiRobot Advance	0.935	3.162	0.300	0.767	−5.262	7.133
Follow-up (month)	−0.035	0.090	−0.390	0.695	−0.211	0.141
Sample size	0.001	0.023	0.050	0.961	−0.044	0.046
Mean age (year)	−0.054	0.103	−0.520	0.601	−0.255	0.148
	R^2^ = 0%; I^2^ = 78.86%					
**Operative Time (min)**						
Bi-planar robot	−24.266	10.722	−2.260	**0.024**	−45.280	−3.251
Universal Robots	18.863	14.914	1.260	0.206	−10.367	48.094
GD-2000	−1.844	15.550	−0.120	0.906	−32.323	28.634
Tianji Robot	−8.777	14.779	−0.590	0.553	−37.743	20.188
TiRobot Advance	−21.372	14.532	−1.470	0.141	−49.856	7.111
Follow-up (month)	0.698	0.458	1.530	0.127	−0.199	1.595
Sample size	0.167	0.139	1.200	0.230	−0.106	0.440
Mean age (year)	0.160	0.508	0.310	0.753	−0.836	1.156
	R^2^ = 33.43%; I^2^ = 97.13%					
**Frequency of Fluroscopy**						
Bi-planar robot	−20.472	3.989	−5.130	**0.000**	−28.290	−12.655
Universal Robots	−32.172	3.941	−8.160	**0.000**	−39.896	−24.447
GD-2000	−4.283	4.806	−0.890	0.373	−13.702	5.137
Tianji Robot	2.493	3.095	0.810	0.421	−3.574	8.559
TiRobot Advance	−0.787	3.234	−0.240	0.808	−7.125	5.552
Follow-up (month)	0.014	0.113	0.130	0.898	−0.207	0.236
Sample size	0.060	0.033	1.830	0.068	−0.004	0.124
Mean age (year)	−0.450	0.175	−2.570	**0.010**	−0.792	−0.107
	R^2^ = 94.67%; I^2^ = 85.30%					
**Frequency of Guide Pin Insertion**						
Bi-planar robot	−3.126	3.980	−0.790	0.432	−10.927	4.675
Universal Robots	−7.283	3.637	−2.000	**0.045**	−14.411	−0.155
GD-2000	4.888	4.797	1.020	0.308	−4.515	14.291
TiRobot Advance	0.913	3.127	0.290	0.770	−5.216	7.041
Follow-up (month)	−0.202	0.180	−1.120	0.262	−0.556	0.151
Sample size	0.013	0.039	0.340	0.738	−0.063	0.089
Mean age (year)	−0.503	0.263	−1.910	0.056	−1.018	0.013
	R^2^ = 44.45%; I^2^ = 92.81%					
**Intraoperative Blood Loss (mL)**						
Bi-planar robot	21.287	14.230	1.500	0.135	−6.604	49.179
Universal Robots	−37.713	14.017	−2.690	**0.007**	−65.186	−10.241
GD-2000	18.924	15.664	1.210	0.227	−11.777	49.625
Tianji Robot	1.532	13.189	0.120	0.908	−24.318	27.381
TiRobot Advance	−4.107	12.578	−0.330	0.744	−28.760	20.546
Follow-up (month)	−0.789	0.487	−1.620	0.105	−1.744	0.165
Sample size	−0.225	0.106	−2.110	**0.035**	−0.433	−0.016
Mean age (year)	−2.980	0.826	−3.610	**0.000**	−4.598	−1.362
	R^2^ = 51.16%; I^2^ = 96.49%					

The reference (comparison) group in the robot type variable was TiRobot. Bold values indicated statistically significant results (*p* < 0.05). SE: standard error; CI: confidence interval; P; *p*-value.

## Data Availability

The analyzed data in this research can be provided by the corresponding author upon reasonable request.

## Supplementary Materials

The following supporting information can be downloaded at https://www.mdpi.com/article/10.3390/jcm13175072/s1. Table S1. The search query employed in each of the searched databases in this systematic review; Figure S1. Leave-one-out sensitivity analysis of union time between robot-assisted and freehand screw placement in patients with femoral neck fractures; Figure S2. Leave-one-out sensitivity analysis of Harris score between robot-assisted and freehand screw placement in patients with femoral neck fractures; Figure S3. Leave-one-out sensitivity analysis of operative time between robot-assisted and freehand screw placement in patients with femoral neck fractures; Figure S4. Leave-one-out sensitivity analysis of frequency of fluoroscopy between robot-assisted and freehand screw placement in patients with femoral neck fractures; Figure S5. Leave-one-out sensitivity analysis of intraoperative fluoroscopy time between robot-assisted and freehand screw placement in patients with femoral neck fractures; Figure S6. Leave-one-out sensitivity analysis of frequency of guide pin insertion between robot-assisted and freehand screw placement in patients with femoral neck fractures; Figure S7. Leave-one-out sensitivity analysis of frequency of intraoperative drilling between robot-assisted and freehand screw placement in patients with femoral neck fractures; Figure S8. Leave-one-out sensitivity analysis of intraoperative blood loss between robot-assisted and freehand screw placement in patients with femoral neck fractures; Figure S9. Leave-one-out sensitivity analysis of postoperative hospitalization time between robot-assisted and freehand screw placement in patients with femoral neck fractures.
