# Supplementary material for: Comparative Efficacy and Safety of Robot-Assisted vs. Freehand Screw Placement in Femoral Neck Fractures: An Updated Systematic Review and Meta-Analysis
Source: J Clin Med. 2024 Aug 27;13(17):5072. doi: 10.3390/jcm13175072 (PMC11396692; doi:10.3390/jcm13175072)
Supplement: Supplementary file 1 [file jcm-13-05072-s001.zip › Table S1.pdf]

Table S1. The search query employed in each of the searched databases in this systematic review

Database	No.	Search Query	Results
**PubMed [Date of search: July 9, 2024]**			
	#1	“Fractures,Bone“[Mesh] OR “Fracture Healing“[Mesh] OR “Fracture Fixation, Intramedullary“[Mesh] OR “Fracture Fixation, Internal“[Mesh] OR “Fracture Fixation“[Mesh] OR “Open Fracture Reduction“[Mesh] OR “Closed Fracture Reduction“[Mesh]	79,883
	#2	"femoral neck"[tiab] OR "femur neck"[tiab]	25,000
	#3	Robot*[tiab] OR “Robotics“[MeSH Terms]	86,806
	#4	#1 AND #2 AND #3	23
**Scopus [Date of search: July 9, 2024]**			
	#1	TITLE-ABS-KEY (Fracture*)	332,770
	#2	TITLE-ABS-KEY ("femoral neck") OR TITLE-ABS-KEY ("femur neck")	37,249
	#3	TITLE-ABS-KEY (Robot*)	89,161
	#4	#1 AND #2 AND #3	79
**Web of Science [Date of search: July 9, 2024]**			
	#1	AB=Fracture*	78,395
	#2	AB="femoral neck" OR AB="femur neck"	6,921
	#3	AB=Robot*	33,720
	#4	#1 AND #2 AND #3	31
**CENTRAL [Date of search: July 9, 2024]**			
	#1	Fracture OR fractures	131,653
	#2	"femoral neck" OR "femur neck"	3,136
	#3	Robot OR robotic OR robotically	9,250
	#4	#1 AND #2 AND #3	3
**Clinicaltrials.gov [Date of search: July 9, 2024]**			
	Condition/disease	Femoral neck fracture	-
	Other terms	-	-
	Intervention/treatment	Robotic	-
	Total	Filters: Completed or With Results	0
**Google Scholar [Date of search: July 9, 2024]**			
	With all of the words	neck fracture robot	-
	With the exact phrase	-	-
	With at least one of the words	femur femoral	-
	Total	According to recent recommendations, only the first 200 records were retreived	200
