# Supplementary figures and images for: Comparative Efficacy and Safety of Robot-Assisted vs. Freehand Screw Placement in Femoral Neck Fractures: An Updated Systematic Review and Meta-Analysis
Source: J Clin Med. 2024 Aug 27;13(17):5072. doi: 10.3390/jcm13175072 (PMC11396692; doi:10.3390/jcm13175072)

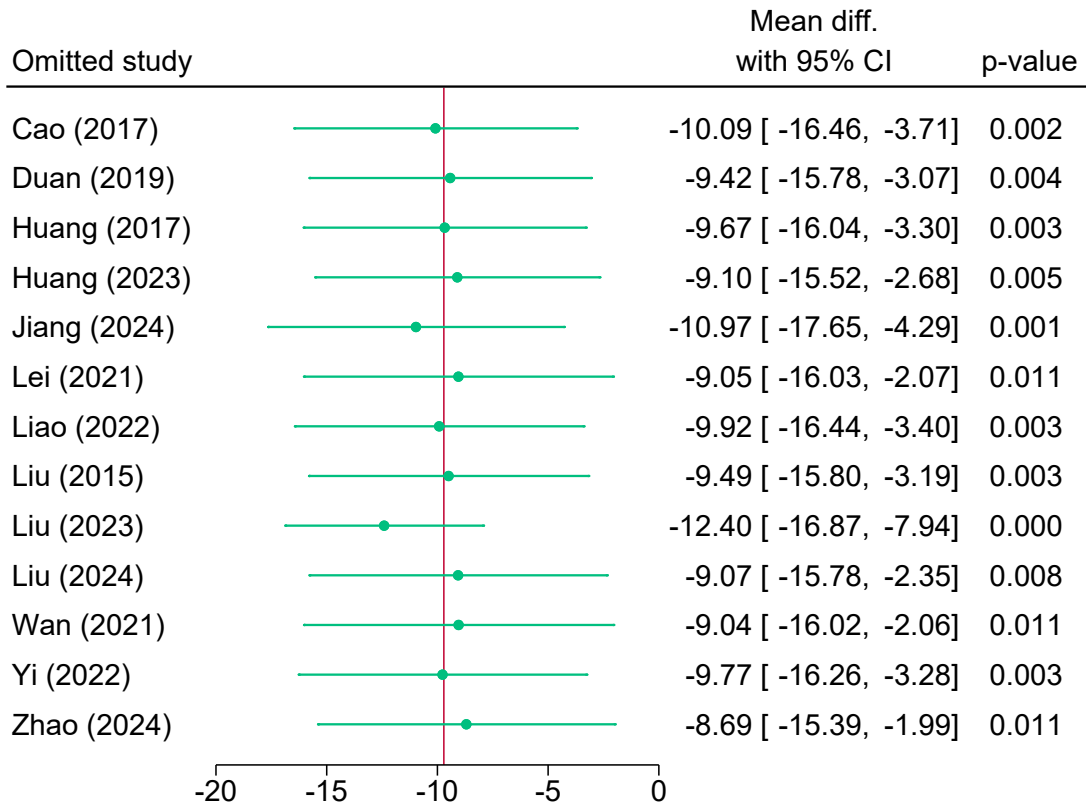

Random-effects REML model

Supplement: Supplementary file 1 [file jcm-13-05072-s001.zip › Figure S1.pdf]

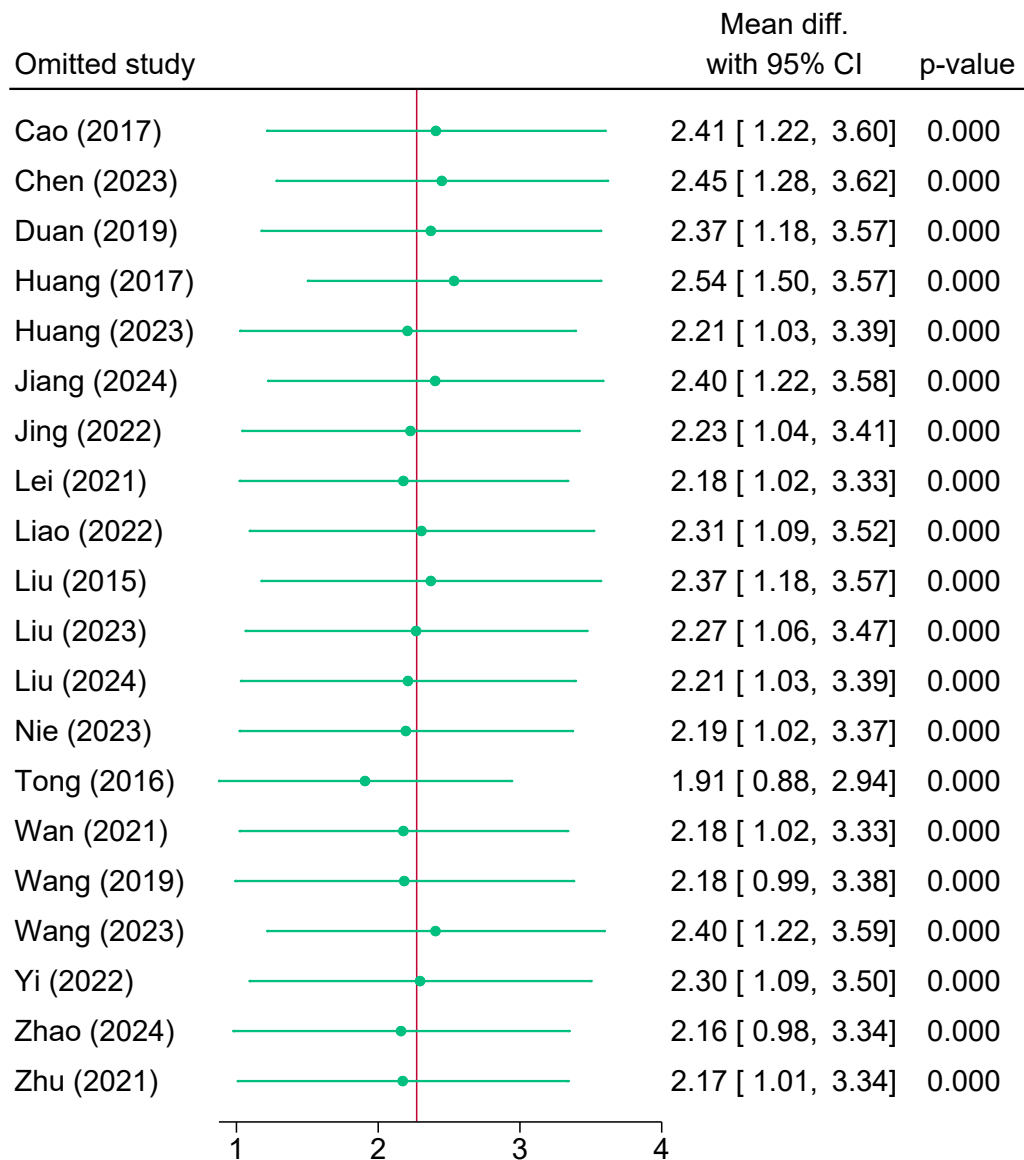

Random-effects REML model

Supplement: Supplementary file 1 [file jcm-13-05072-s001.zip › Figure S2.pdf]

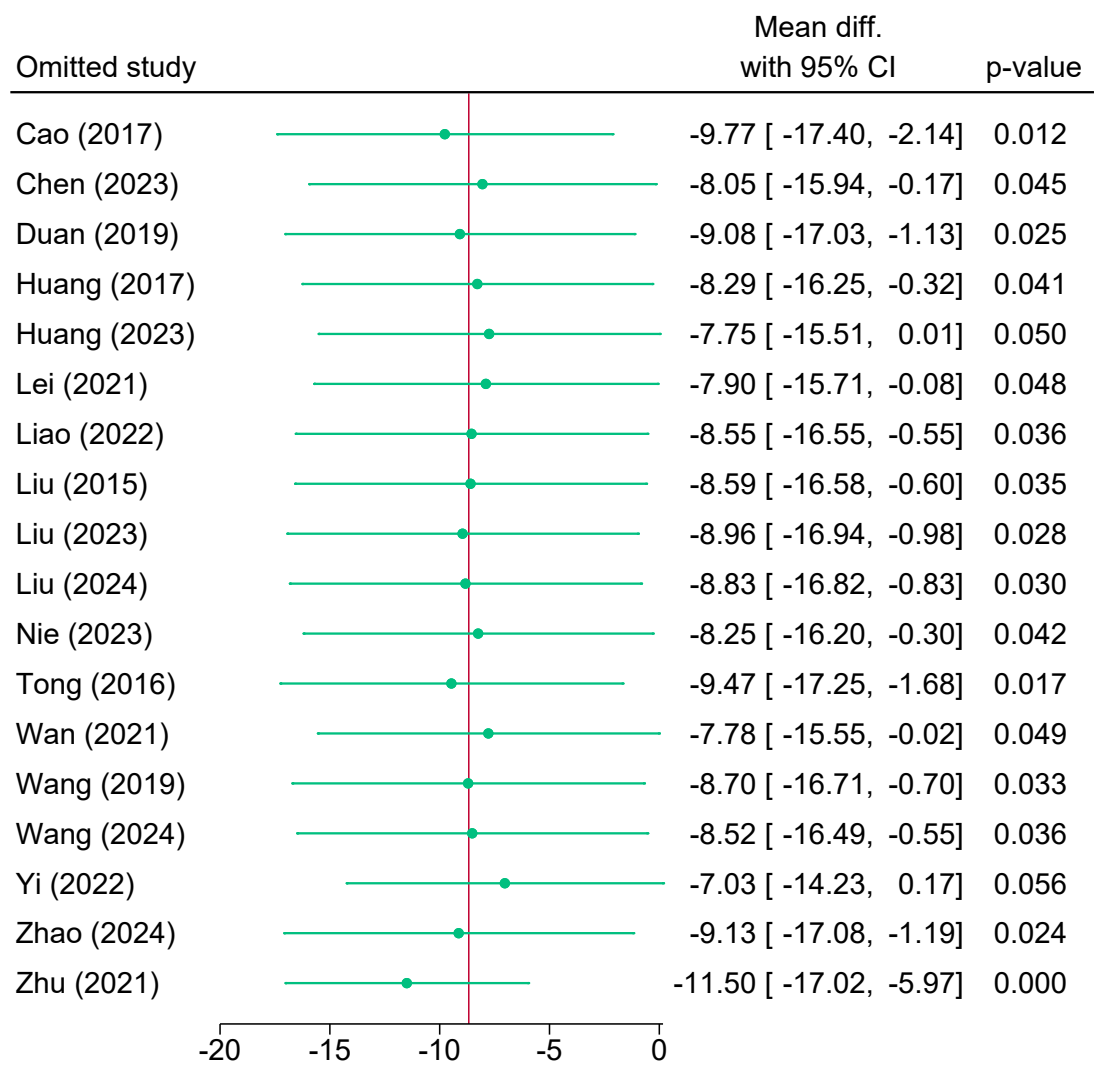

Random-effects REML model

Supplement: Supplementary file 1 [file jcm-13-05072-s001.zip › Figure S3.pdf]

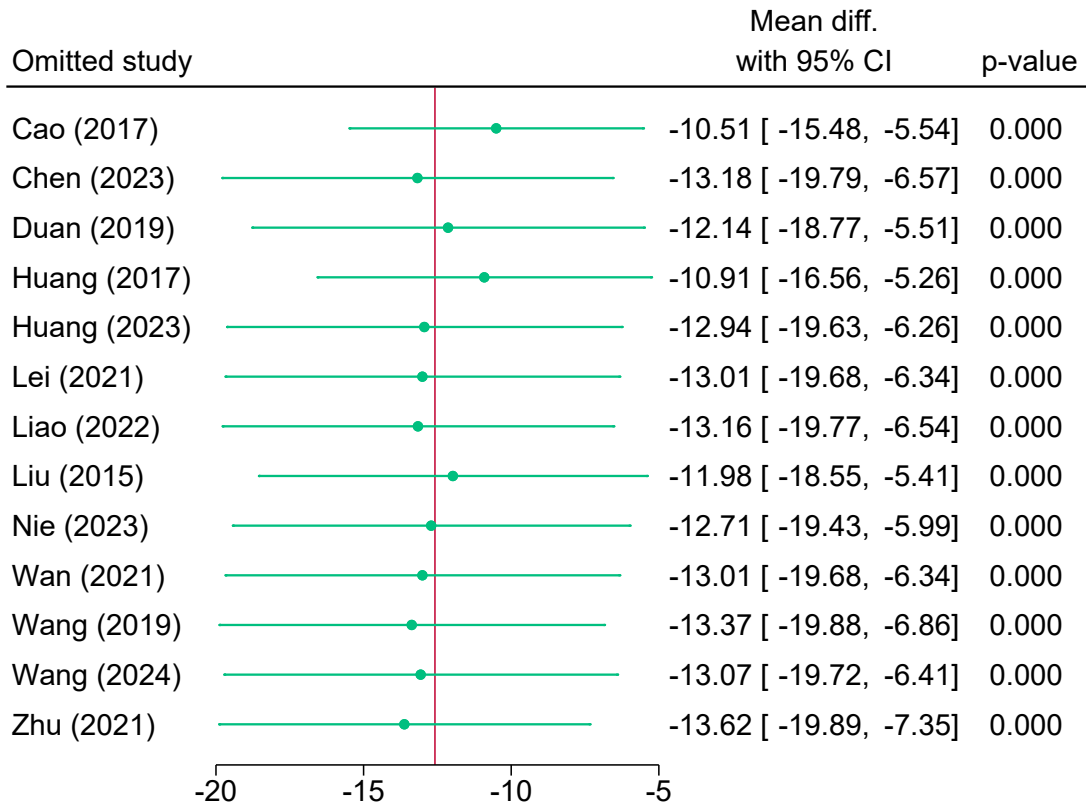

Random-effects REML model

Supplement: Supplementary file 1 [file jcm-13-05072-s001.zip › Figure S4.pdf]

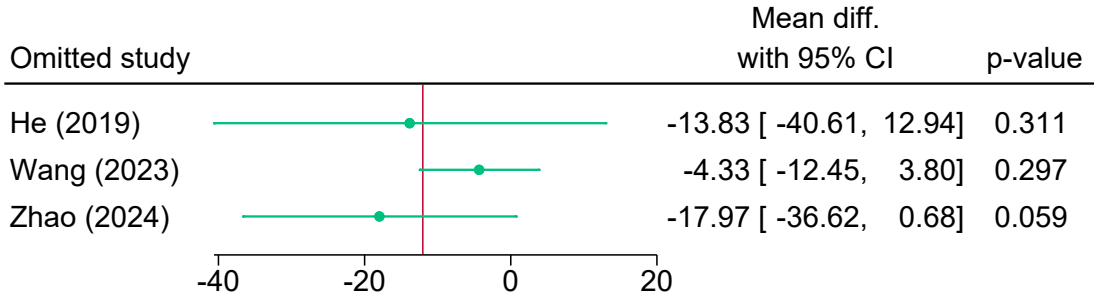

Random-effects REML model

Supplement: Supplementary file 1 [file jcm-13-05072-s001.zip › Figure S5.pdf]

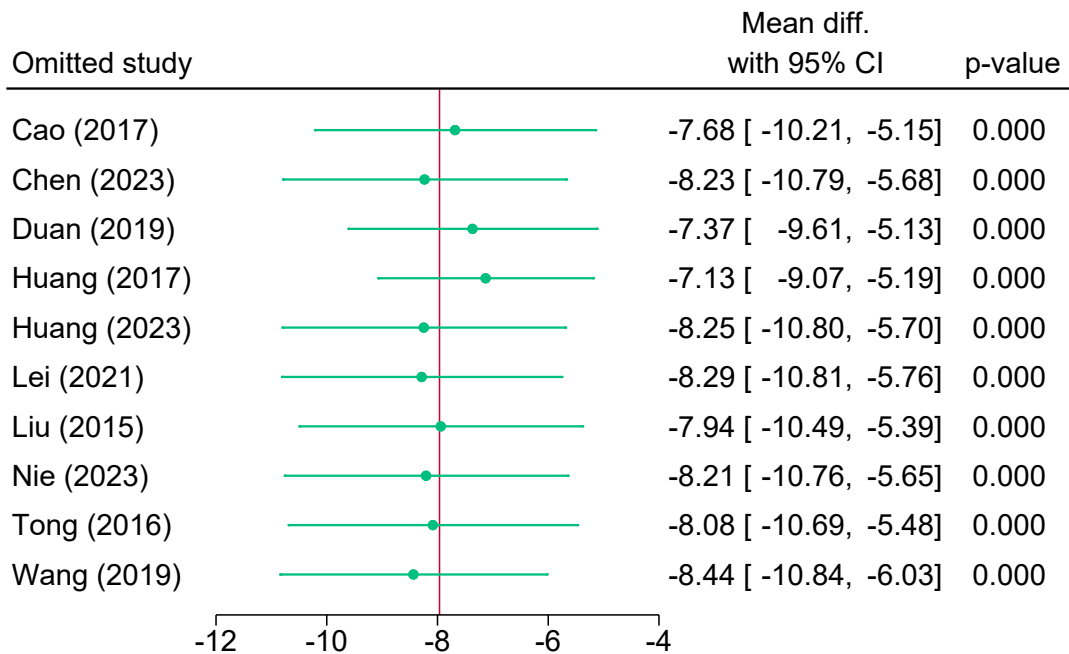

Random-effects REML model

Supplement: Supplementary file 1 [file jcm-13-05072-s001.zip › Figure S6.pdf]

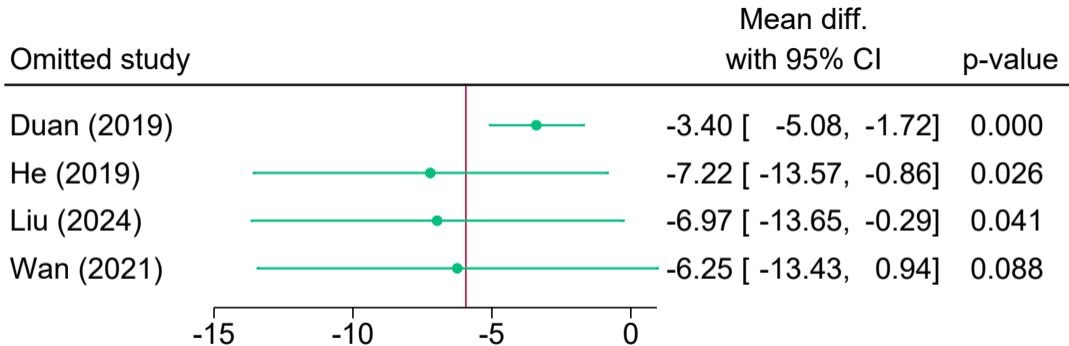

Random-effects REML model

Supplement: Supplementary file 1 [file jcm-13-05072-s001.zip › Figure S7.pdf]

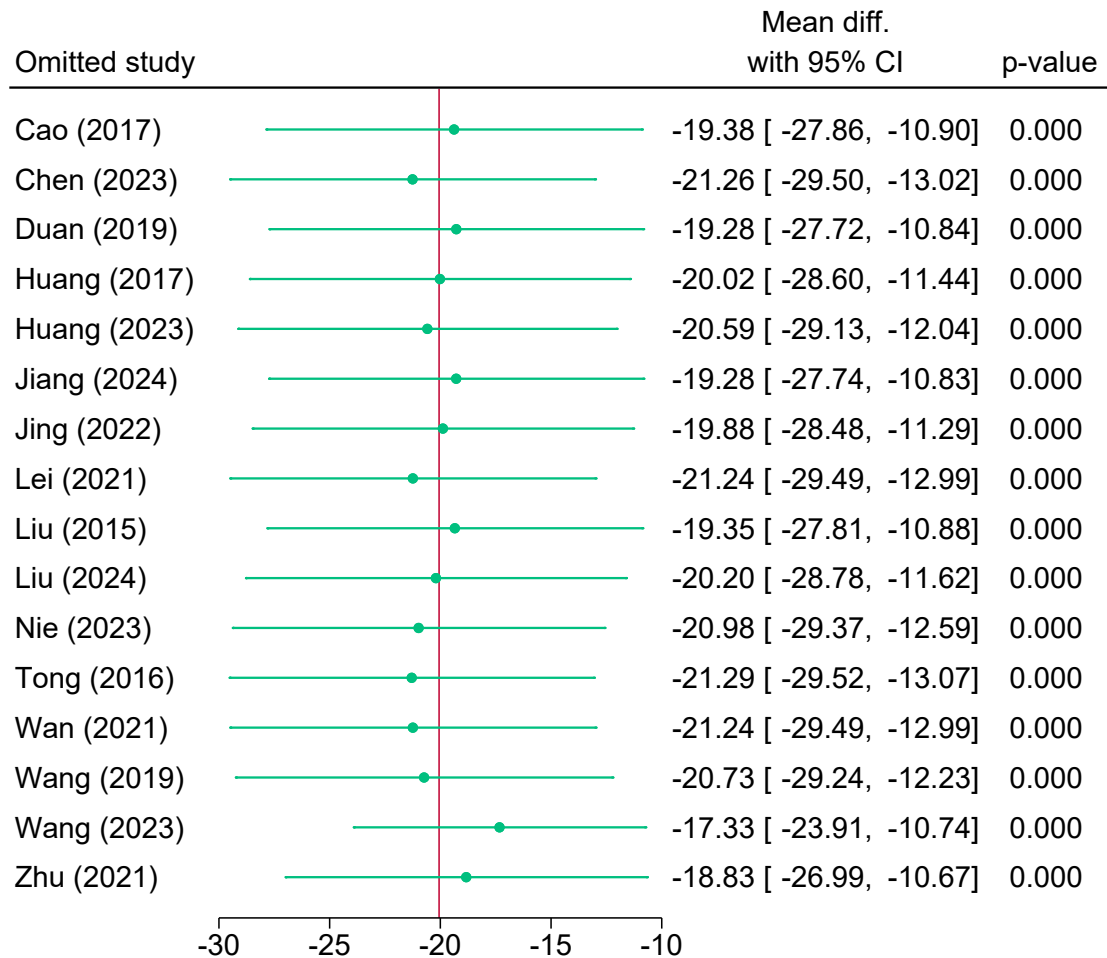

Random-effects REML model

Supplement: Supplementary file 1 [file jcm-13-05072-s001.zip › Figure S8.pdf]

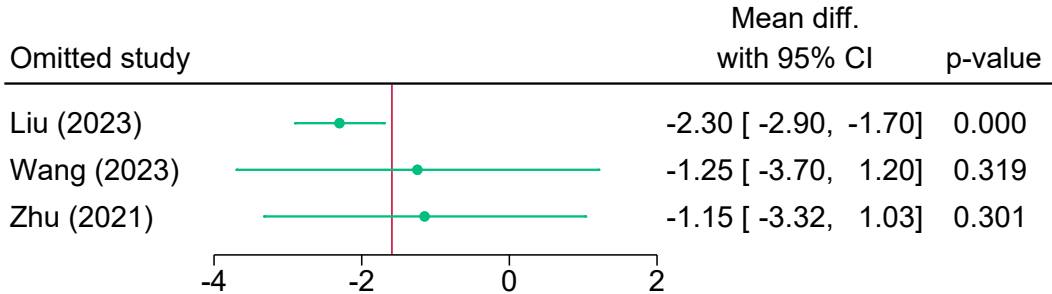

Random-effects REML model

Supplement: Supplementary file 1 [file jcm-13-05072-s001.zip › Figure S9.pdf]
